# The Effect of Non-Invasive Brain Stimulation on Running Performance and Inertial Measurement Unit-Derived Spatiotemporal Parameters in Endurance-Trained Runners

**DOI:** 10.3390/s26175390

**Published:** 2026-08-26

**Authors:** Isabella Sierra, Yiyang Chen, Gleydciane Alexandre Fernandes, Henri Lajeunesse, Julien Clouette, Alexandra Potvin-Desrochers, Jenna C. Gibbs, Julie N. Côté, Fabien A. Basset, Caroline Paquette

**Affiliations:** 1 Department of Kinesiology and Physical Education, McGill University, Montreal, QC H2W 1S4, Canada; yiyang.chen@memphis.edu (Y.C.); ciane.alexandrefernandes@mail.mcgill.ca (G.A.F.); henri.lajeunesse@mail.mcgill.ca (H.L.); julien.clouette@mail.mcgill.ca (J.C.); alexandra.potvin-desrochers@mcgill.ca (A.P.-D.); jenna.gibbs@mcgill.ca (J.C.G.); julie.cote2@mcgill.ca (J.N.C.); caroline.paquette@mcgill.ca (C.P.); 2 Jewish Rehabilitation Hospital Site of Santé Québec Laval and Research Site of the Montreal Centre for Interdisciplinary Research in Rehabilitation (CRIR), Laval, QC H7V 1R2, Canada; 3 College of Health and Human Sciences, University of Memphis, Memphis, TN 38152, USA; 4 Centre de Recherche, Centre de Recherche, Santé Québec Outaouais, Gatineau, QC J8T 4J3, Canada; 5 Metabolic Disorders and Complications Program, Research Institute of the McGill University Health Centre, Montreal, QC H4A 3J1, Canada; 6 School of Human Kinetics and Recreation, Memorial University of Newfoundland, St. John’s, NL A1C 5S7, Canada; fbasset@mun.ca

**Keywords:** neuromodulation, running performance, spatiotemporal parameters, inertial measurement unit

## Abstract

Integrating wearable motion sensing with neuromodulation may improve understanding of how alterations in neural excitability influence running performance and biomechanics. This study investigated whether intermittent theta burst stimulation (iTBS) applied to the primary motor cortex (M1), dorsolateral prefrontal cortex (DLPFC), or both regions influences running performance and sensor-derived spatiotemporal parameters during a 3000 m time-trial run. Ten endurance-trained runners (7 males) completed four stimulation conditions (M1, DLPFC, M1 + DLPFC, and sham) in a randomized, sham-controlled, repeated-measures crossover design. Running performance and spatiotemporal gait parameters were continuously monitored using wearable inertial measurement units (IMUs), with analyses conducted across the initial, steady-state, and final acceleration phases of the run. The M1 + DLPFC condition resulted in the fastest mean completion time, averaging approximately three seconds faster than sham. However, these differences were not statistically significant. Sensor-derived biomechanical measures revealed significantly higher running speeds and alterations in stride time and step frequency during the initial phase following combined stimulation compared with the other conditions. Ratings of perceived exertion and spatiotemporal variability did not differ between stimulation conditions. These findings demonstrate the utility of wearable IMUs for detecting subtle phase-specific changes in running biomechanics and suggest that combined stimulation of motor and cognitive control regions may influence early-stage running performance, warranting further investigation in larger cohorts. As the complete sample consisted of only ten runners, these findings are preliminary and require confirmation in a larger sample size.

## 1. Introduction

Utilizing non-invasive brain stimulation (NIBS) to increase cortical excitability has been explored as a potential method to enhance endurance running performance. Although several brain regions are associated with factors relevant to athletic performance, the primary motor cortex (M1) and dorsolateral prefrontal cortex (DLPFC) are among the most commonly explored regions. The M1 is responsible for voluntary muscle contraction and the execution of movements [1]. The M1 has a somatotopic organization, meaning specific areas of the cortex correspond to the control of particular muscles [2]. Muscle contraction is driven by motor neurons in the spinal cord, collectively known as the motor neuron pool, which send signals from the brain to the muscles [3]. During endurance exercise, the motor neuron pool experiences decreased excitability, which results in reduced muscle capacity and contributes to fatigue [4]. Increasing the excitability of M1 may help to slow this decrease in neural drive that occurs due to exercise-related fatigue [5]. Increased neural drive could lead to greater muscle output, potentially allowing runners to produce greater force to maintain higher intensity exercise for longer durations.

Failure to sustain sufficient muscle output alone is not the sole factor determining exercise termination, as studies have shown that activity in the prefrontal cortex decreased just prior to exercise termination [6]. The DLPFC, specifically, is involved in pain perception, motivation and cognitive control, all of which influence endurance exercise [5]. Previous studies suggest that an athlete’s ability to tolerate exercise-induced pain is associated with performance levels [7]. Additionally, increased excitability of the left DLPFC has been linked to reduced rating of perceived exertion (RPE) and a lower degree of pain perception [5], which may allow athletes to endure discomfort caused by exercise for longer periods of time.

The limited research that has examined applying NIBS to either the M1 or DLPFC in the context of running performance has produced mixed results. The discrepancy in findings may be partially attributed to differences in stimulation protocols. The majority of studies have used a type of NIBS called transcranial direct current stimulation (tDCS) [8,9,10]. Although tDCS is cost-effective and portable, the lack of uniform protocols makes it challenging to compare studies directly. For instance, in the few studies that have examined running performance after excitatory M1 stimulation, variances existed in stimulation intensity, electrode size, and electrode placement.

To date, only one study has examined tDCS in an overground running setting. Following stimulation, Dos Santos et al. [8] found a decrease in overall time to complete a 5000 m run on an indoor track compared to a sham stimulation group. However, this study used a between-subjects design with no baseline testing for either group. Therefore, the difference in time to complete the time-trial run could be explained by differing performance levels in individuals, as the performance levels of each group were not reported. Of the studies that assessed running performance on a treadmill, only one reported an increase in time to exhaustion [9], with the other two finding no significant difference in exercise tolerance compared to sham [10,11]. Despite all three studies recruiting male participants with similar values of maximal oxygen uptake ($\overset{\cdot }{V}$O_2_max), further discrepancies were evident in sample size and treadmill protocols. These methodological differences may influence how well the results reflect improvements in actual running performance. Although fixed-speed treadmill protocols offer greater control, they prevent runners from using natural pacing strategies that are typically observed when individuals can adjust their own speed, such as on a self-paced treadmill or during overground running. Even on self-paced treadmills, however, the altered visual feedback can make it challenging for runners to pace themselves naturally. This limitation of treadmill testing could limit the potential effects of increased cortical excitability that vary across different phases of a run.

The optimal values of several spatiotemporal parameters may reflect differences in running style rather than differences in performance level [12]. For example, while some studies have associated lower step frequency (SF) with better performance [13], others have reported superior performance among runners with higher SF [14]. Similar inconsistencies have been observed for duty factor (DF), defined as the proportion of contact time (CT) within a complete gait cycle. A lower DF is characterized by a shorter CT and a longer flight phase. Although longer flight phases have been linked to improved performance [13], the accompanying reduction in CT may require greater force production, which could increase physiological demands and potentially impair performance. Therefore, the absolute magnitude of these parameters may not be a reliable indicator of performance. Instead, examining how they change in response to increased excitability may provide valuable insight into the gait adaptations adopted by runners.

Excessive variability in these spatiotemporal parameters, particularly when induced by fatigue, can impair performance [15]. While spatiotemporal parameters refer to the average or mean values of movement characteristics over time and space, spatiotemporal variability reflects the consistency of these parameters across strides, often quantified using metrics such as the coefficient of variation (CV) or standard deviation (SD). Although a certain amount of variability in running is inevitable [16], higher-level runners typically exhibit more consistent biomechanics and reduced variability compared to lower-level runners [17]. More experienced runners have shown reduced variability in SF [18], CT [19], and stride time (ST) [20]. Collecting information on both spatiotemporal parameters and spatiotemporal variability throughout a run may provide further meaningful objective insight compared to time alone. Inertial measurement units (IMUs) allow continuous monitoring of spatiotemporal running characteristics during overground exercise with little disruption to natural movement patterns. Incorporating sensor-derived biomechanical metrics may improve understanding of the mechanisms through which increases in cortical excitability influence endurance running performance.

Spatiotemporal parameters and variability have not been used as outcome measures in studies that specifically increase cortical excitability to enhance endurance performance. However, studies on healthy young individuals performing walking tasks have shown increased stride length (SL) and speed [21] after M1 stimulation and reduced ST variability [22] during dual-tasking after DLPFC stimulation. This effect of reduced variability could be attributed to the DLPFC’s role in cognitive functions such as task prioritization [5], which in the context of middle-distance running, could be beneficial for pacing strategies and regulation of effort.

Although there is interest in both M1 and DLPFC separately, few studies have explored stimulating both regions together to improve multiple aspects of performance at the same time. One study from Schneider et al. [23] found that stimulating both M1 and DLPFC in healthy older adults led to increased gait speed and reduced ST variability during dual-tasking. Based on previous research, it could be assumed that the increase in speed was driven by M1 stimulation, while the reduction in variability resulted from DLPFC stimulation. However, as the study did not stimulate each region separately, it is impossible to determine whether these effects were due to the combined influence of both areas or if one region was primarily responsible.

Since M1 stimulation could lead to greater muscle activation, improving physical performance, and DLPFC stimulation may reduce perceived exertion and variability and improve task prioritization, increasing excitability in both the M1 and DLPFC has the potential to enhance performance more so than either area alone. Accordingly, this study examined how targeting M1, DLPFC, or both influences total time, speed, RPE, and spatiotemporal parameters of ST, SF, SL, and DF, as well as their variability throughout a 3000 m time-trial. Since only ten runners completed all four sessions, this study should be considered exploratory, with all analyses acting as initial tests of the following hypotheses:

1. The M1 only and M1 + DLPFC conditions would produce faster total times to complete the 3000 m time-trial run and faster lap speeds compared to stimulation of the DLPFC only or sham stimulation.
2. Runners would show decreased RPE in the DLPFC only and M1 + DLPFC conditions throughout the entirety of the 3000 m time-trial, compared to stimulation of M1 only or sham stimulation.
3. Variability of ST, DF, SF and SL would increase throughout the run; however, stimulation of the DLPFC only and M1 + DLPFC conditions would mitigate the increase in variability more so than stimulation of the M1 only or sham stimulation.

## 2. Materials and Methods

### 2.1. Participants

This study was part of a larger investigation on measuring multilevel effects of increasing excitability on the M1 and DLPFC and running performance [24]. Thirty-seven runners signed a consent form for the study; however, only 23 completed all sessions. From these 23 runners, exclusions were made due to inconsistent performance due to illness, failure to adhere to provided guidelines, incorrect intensity of iTBS applied due to discomfort, or incomplete kinematic data for all four sessions. The final sample for this study included ten endurance-trained runners (3 females, 7 males, mean age = 26 years, SD = 7, range = 18–41 years; Table 1).

Runners were recruited via outreach to the institutional varsity cross country and track teams, as well as coaches at nearby schools and universities, and postings in running groups on social media. Inclusion criteria required enrolment in an endurance running training program, participation in competitive running events for at least 2 years preceding the study, specialization in running events of 3000 m or longer, and a training volume of >30 km/week. Exclusion criteria included musculoskeletal injury in the year preceding the study, use of central nervous system-acting medications such as antidepressants, and contraindications for TMS and MRI such as metallic hardware or fragments in the head, cochlear implants, deep brain stimulators, pacemakers, history of seizures, and claustrophobia [25]. All runners provided written informed consent according to the McGill Faculty of Medicine Institutional Review Board regulations and the Declaration of Helsinki.

### 2.2. Experimental Protocol

**Figure 1:**
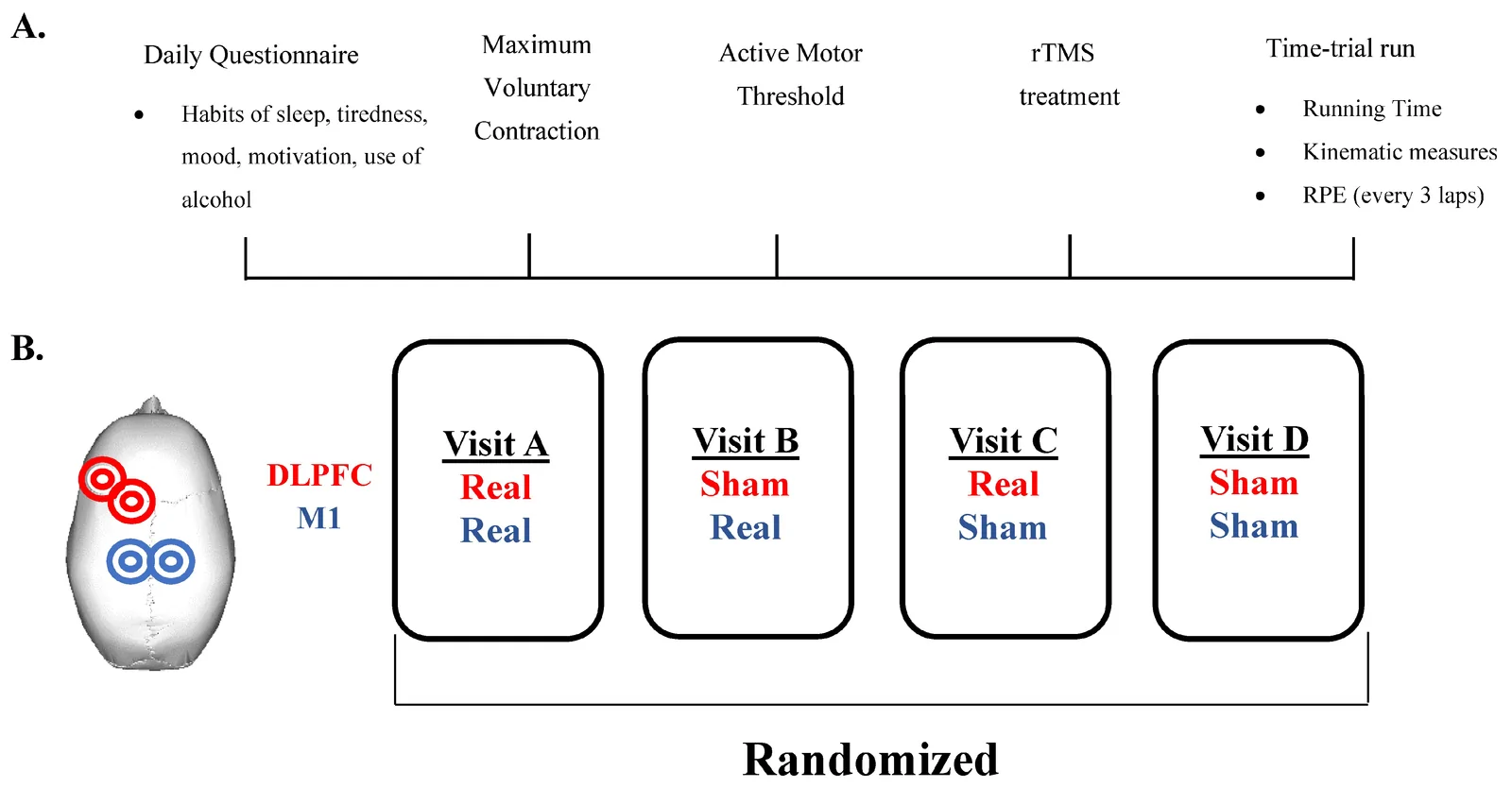
(**A**) Timeline of experimental sessions and measures taken. (**B**) The four rTMS treatments. Runners first received real or sham stimulation of the M1 (stimulation location shown in blue), then received real or sham stimulation over the left DLPFC (shown in red). Order of sessions was randomized and sessions were spaced at least 72 h apart.

Runners visited the laboratory on five occasions: an initial visit followed by four experimental visits. The initial session was used to acquire structural magnetic resonance imaging (MRI), collect runner training profiles and general health habits, familiarize runners with the experimental procedures, set individual TMS stimulation target locations to be used in the experimental visits, and perform a $\overset{\cdot }{V}$O_2_max test. During the four experimental visits (Figure 1), runners were administered one of four iTBS conditions shortly before performing an all-out 3000 m time-trial run.

### 2.3. Initial Session

MRI was acquired from runners to allow for precise coil placement of the DLPFC, which has no direct TMS output. The MRI image was acquired on a Siemens 3T (Siemens, Knoxville, TN, USA) at the McConnell Brain Imaging Centre of the Montreal Neurological Institute (MNI; echo time = 2.96 ms; repetition time = 2300 ms, flip angle = 9°, 192 slices, voxel size = 1 mm^3^ isotropic).

Stimulation points for M1 TA were identified using a Magstim 200^2^ TMS machine (Magstim©, Whitland, UK) by delivering single monophasic pulses during hotspot mapping. For bilateral M1 TA stimulation, the optimal location for TMS was based on the motor responses. The hotspot was identified by mapping the scalp area that produced the greatest response to TMS stimulation. Responses were measured by electromyography (EMG) and required to be similar in magnitude in the right and left TA muscles. The EMG set-up was the same for the initial and experimental sessions with two 2.5 cm × 2.5 cm Ag/AgCl surface gel electrodes (Biopac EL504, Biopac Systems, Inc., Goleta, CA, USA) placed on each of the right and left TA muscle bellies per the SENIAM standard [26] along with a ground electrode at the patella. EMG signals were recorded with a Biopac MP150 acquisition system (Biopac Systems, Inc., Goleta, CA, USA), sampled at 5 kHz on a 16-bit analog-to-digital board, amplified and bandpass filtered (10–2000 Hz). Pulses were delivered to the motor cortex region corresponding to the bilateral TA, located in the interhemispheric fissure anterior to the central sulcus. A 60 mm dome coil (Jaltron LLC, Lexington, MA, USA) was placed on top of the runner’s head in a posterior–anterior (PA) orientation, with the coil current directed anteriorly, and the location that elicited the largest and most consistent (between trials and between right and left TA) motor-evoked potentials (MEPs) was marked in Brainsight (Brainsight neuronavigation system; Rogue Research Inc., Montreal, QC, Canada). The location was the M1 stimulation target of the experimental sessions.

The left DLPFC location was specified by MNI coordinates [27] and marked in Brainsight (x = −50, y = 30, z = 36). The stimulation intensity for the non-motor DLPFC target was based on the resting motor threshold of the M1 of the left first dorsal interosseous (FDI). This region was used because it is a motor region that lies at a similar depth as the DLPFC [28]. Pulses were delivered over the left M1 FDI region with a 50 mm figure of eight coil (Magstim Company, Whitland, UK) placed tangential to the runner’s head in a PA orientation 45 degrees from midline. The location eliciting the largest and most consistent MEPs was marked in Brainsight.

Lastly, $\overset{\cdot }{V}$O_2_max was measured via an incremental treadmill test modified from Leger and Boucher [29] to assess cardiorespiratory fitness of the runner. The procedure began with the runner standing still and breathing normally for two minutes to obtain baseline metabolism. To begin the test, runners warmed up for 5 min on the treadmill at a self-selected pace. Following the warm-up, the test started at a speed of 10 km/h for females and 12 km/h for males. The speed increased by 1 km/h every 2 min until volitional exhaustion was reached [29]. After a five-minute rest, runners then ran at 105% of the final speed reached in the initial test until volitional exhaustion to verify that maximal effort was reached.

### 2.4. Experimental Sessions

The four experimental conditions were given in randomized order, and runners were blinded to the condition they received. Stimulation of the M1 was always delivered before DLPFC stimulation. TMS responses were recorded in the bilateral tibialis anterior (TA) muscles, as they are known to play a crucial role in gait and balance [30]. Sessions were typically scheduled one week apart, with a minimum separation of 72 h to avoid carry-over effects.

At the start of each experimental session, a questionnaire was administered to document runners’ previous 24 h regarding sleep, tiredness, mood, hunger, motivation, and use of alcohol or drugs. Stimulation intensity was set based on active motor threshold (AMT). Maximum voluntary contraction (MVC) was acquired to standardize active muscle contraction when finding motor threshold. Visual feedback of muscle contraction was provided on a monitor placed in front of the runners displaying EMG activity with a target set at ±20% MVC, so that runners could maintain this contraction as pulses were delivered to the target. AMT was determined as the lowest possible stimulation intensity that could elicit an MEP. Intensities for the TMS were set to 80% of the AMT.

MVC for the TA was determined by having the runner place both feet under a slanted wooden board and instructing them to dorsiflex using their TA muscles as hard as possible for about three seconds. A researcher stood on top of the board to provide resistance. Runners repeated this dorsiflexion three times, and the EMG output from each foot was separately recorded. The largest EMG value from the right foot and the largest value from the left foot were compared. The smaller of these two values was taken as the runner’s MVC. The MVC measurement was then repeated for the FDI muscle, with runners performing an isometric contraction by abducting their index finger against a wooden stick while keeping their arm placed on top of the chair’s armrest. The largest EMG value of the three trials was taken as the runner’s MVC for the FDI.

**Figure 2:**
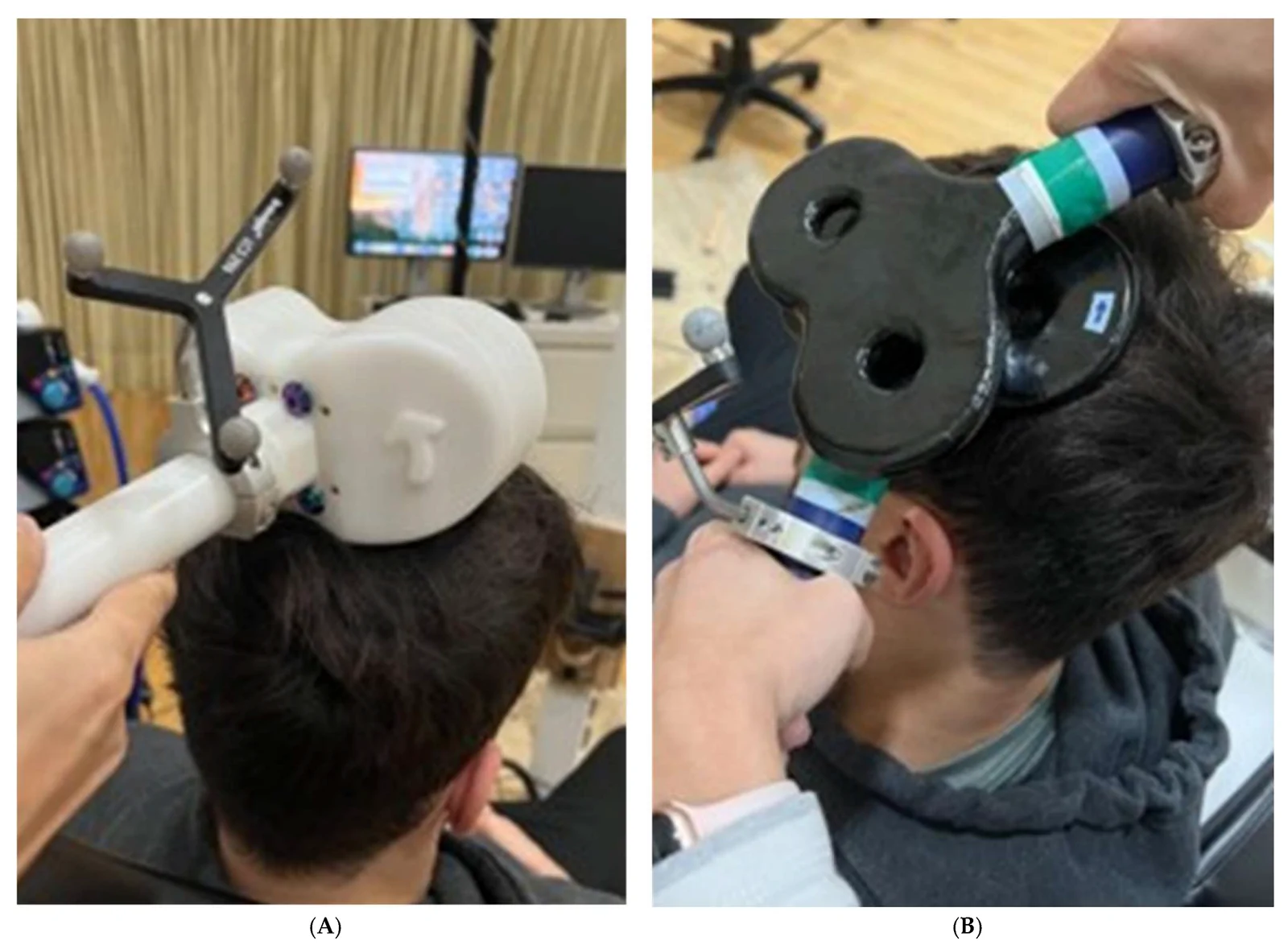
Placement of coils for sham stimulation is shown. (**A**) shows the dome coil (M1 stimulation) rotated 90 degrees to the side. (**B**) shows the primary figure of eight coil (DLPFC stimulation) flipped and placed over a secondary figure of eight coil which was not connected to the stimulator.

The lowest possible intensity that could produce 10 out of 20 MEPs, while runners maintained the 20% MVIC contraction, was determined to be the AMT, and multiplied by 0.8 to obtain the stimulation intensity for bilateral TA and DLPFC stimulation.31 Excitatory TBS was given at a rate of 50 Hz of stimuli at an interval of 5 Hz in 2 s periods, repeated every 10 s for 192 s total [31]. Real or sham stimulation of the M1 was performed first, and then immediately after, real or sham stimulation was delivered to the DLPFC. For sham treatment of the M1, the dome coil was rotated at 90 degrees to the side of the coil, so pulses were directed into the brain (Figure 2A). For sham treatment of the DLPFC, a secondary figure of eight coil was disconnected from the stimulator and placed over the DLPFC hotspot (Figure 2B). The original figure of eight coil was flipped then placed on top of the secondary coil [28]. These methods provided a similar sensation on the scalp and the same auditory stimulus. Sham stimulation was delivered at a lower intensity of 40% of the stimulation intensity.

After stimulation, runners jogged to a 200 m indoor track about two minutes from the stimulation laboratory. To measure movement kinematics and spatiotemporal parameters, seven APDM Opal movement Sensors (APDM Wearable Technologies Inc., Portland, OR, USA) were then placed on the runner on the sternum, lumbar, right wrist, right upper leg, right lower leg, and right and left foot. Acceleration data from the sensors was collected through APDM Moveo Explorer software v1.0.0.202206 (APDM Wearable Technologies Inc., Portland, OR, USA) at a frequency of 128 Hz.

Next, runners performed a 10 min warm-up. The runners self-selected their warm-up routine in their first experimental session. The warm-up patterns were documented so they could be repeated in all subsequent experimental sessions. Runners were instructed to give maximal effort while running in the first lane of the indoor track (banked at an 11-degree angle) for 15 laps. A video camera was placed at the start line to record each instance they crossed the line, and a researcher stood at the start line with a stopwatch to provide runners with their lap times. Every 600 m (three laps), runners verbally reported RPE scores using the Borg 6–20 scale [32]. Runners were informed when they had two laps remaining and again when they had one lap to go. No other feedback or encouragement was provided. Runners were not given their total time until all four experimental sessions were completed.

### 2.5. Data Processing

Data recorded from the APDM sensors were imported into MATLAB R2024a (MathWorks, Natick, MA, USA) for processing. The forward acceleration of the lumbar sensor was utilized to calculate angular velocity. A threshold of 80% of the median was used to separate straight running from turning events [33] along the curved portion of the track, and turning events were eliminated. Analysis was then performed only on the straight-running segment on the side of the track closest to the APDM receivers. Since most of the runners were right foot dominant, gait events were determined for the right leg using the right foot accelerometer.

Foot strikes and toe-offs were identified by analyzing the derivative of the filtered right-foot acceleration in the forward running direction. Instances where acceleration changed direction were identified. Events showing deceleration (negative peaks) were determined to be ground contact and labeled as foot strike. Positive peaks were labeled as toe-off. A complete gait cycle was defined as the time between two consecutive right-foot strikes [34]. The final lap was omitted, as the runners crossed the finish line before completing the entire length of the straightaway.

### 2.6. Outcomes

Total time recorded by the stopwatch was verified against start-line video data. Expected time-trial performance time was calculated by converting the initial session’s $\overset{\cdot }{V}$O_2_max to METS to estimate running speed (km/h), which was then converted to meters per second. The total distance of 3000 m was divided by speed, yielding a predicted 3000 m time-trial run time in seconds [29]. This equation predicted the total time of the runner for the 3000 m time-trial run based on their measured $\overset{\cdot }{V}$O_2_max. Percentage of expected time was calculated by subtracting the expected time from the measured time, dividing by the expected time and then multiplied by 100%. Lap speed was calculated by dividing the lap distance (200 m) by lap time (seconds). Five measurements of RPE were reported by the runner every three laps of the time-trial run.

Stride time was defined as the duration of a full gait cycle, from one right heel strike to the next. Duty factor was calculated as CT divided by ST, then multiplied by 100%. Stride frequency was calculated as 60 divided by CT, yielding the number of strides per minute, then multiplied by two to account for both steps. SF was normalized by multiplying by the distance from the right anterior superior iliac spine to the medial malleolus [18]. Stride length referred to the length between one right foot strike and the next consecutive right foot strike. It was calculated as running speed (m/s) during the straight portion multiplied by CT and normalized using the same method as SF. The CV for ST, DF, SF, and SL was calculated for each lap as the given parameter’s standard deviation across all gait cycles in a lap, divided by the mean gait cycle, multiplied by 100%.

Three phases of the run were determined by graphing speed data over the course of the run. The steady state phase, where speed was relatively stable with no major increases or decreases, was found to be in laps 4 to 12. The initial phase (1–3) and the final acceleration phase (laps 13–15) were faster than the steady state phase. When comparing values of speed, spatiotemporal parameter and spatiotemporal variability, lap 1 was used as the initial lap, lap 12 was used as the steady state lap, and lap 15 for speed and lap 14 for spatiotemporal parameters and variability were used as the final acceleration lap. When comparing percent change, the change in the initial phase was from lap 1 to lap 3, the change in the steady state phase was between laps 4 and 12 and change in the final acceleration phase was between laps 13 and 15 for speed and between laps 13 and 14 for spatiotemporal parameters and variability.

### 2.7. Statistical Analysis

All data were analyzed using SPSS 29 (IBM, New York, NY, USA). Normality was assessed with Shapiro–Wilk tests (*p* > 0.05), and Mauchly’s test assessed sphericity (*p* > 0.05). When sphericity was violated, Greenhouse-Geisser corrections were applied. Effect sizes are reported using partial eta squared (η^2^_p_) and Cohen’s d. Thresholds for interpreting effect sizes are small (η^2^_p_ = 0.01, d = 0.2), medium (η^2^_p_ = 0.06, d = 0.5), and large (η^2^_p_ = 0.14, d = 0.8).

Two one-way repeated-measures analyses of variance (RM-ANOVA) on the condition factor (4 levels) were used to compare total running time and percentage of the runner’s actual time to their expected time. Nine two-way RM-ANOVAs (conditions-4 levels: Sham, M1 only, DLPFC only, M1 + DLPFC X lap-3 levels: Initial, steady state, final acceleration) were used to compare the values of the following factors: lap speed, spatiotemporal parameters (ST, SF, SL, DF), and CV of spatiotemporal parameters. The normality assumption was violated for percent change in lap speed, ST, SF and SL and CV of ST, SF and SL. These factors were assessed with seven non-parametric Friedman’s tests. In the event of significance, Wilcoxon signed rank tests were performed to compare factors across conditions. The normality assumption was not violated for percent change in DF or CV of DF, and two additional two-way RM-ANOVA was done to assess these factors (conditions-4 levels: Sham, M1 only, DLPFC only, M1 + DLPFC X phase-3 levels: Initial, steady state, final acceleration). One two-way RM-ANOVA (conditions-4 levels: Sham, M1 only, DLPFC only, M1 + DLPFC X lap-5 levels: laps 3, 6, 9, 12 and 15) was used to assess RPE.

## 3. Results

### 3.1. Running Performance

**Figure 3:**
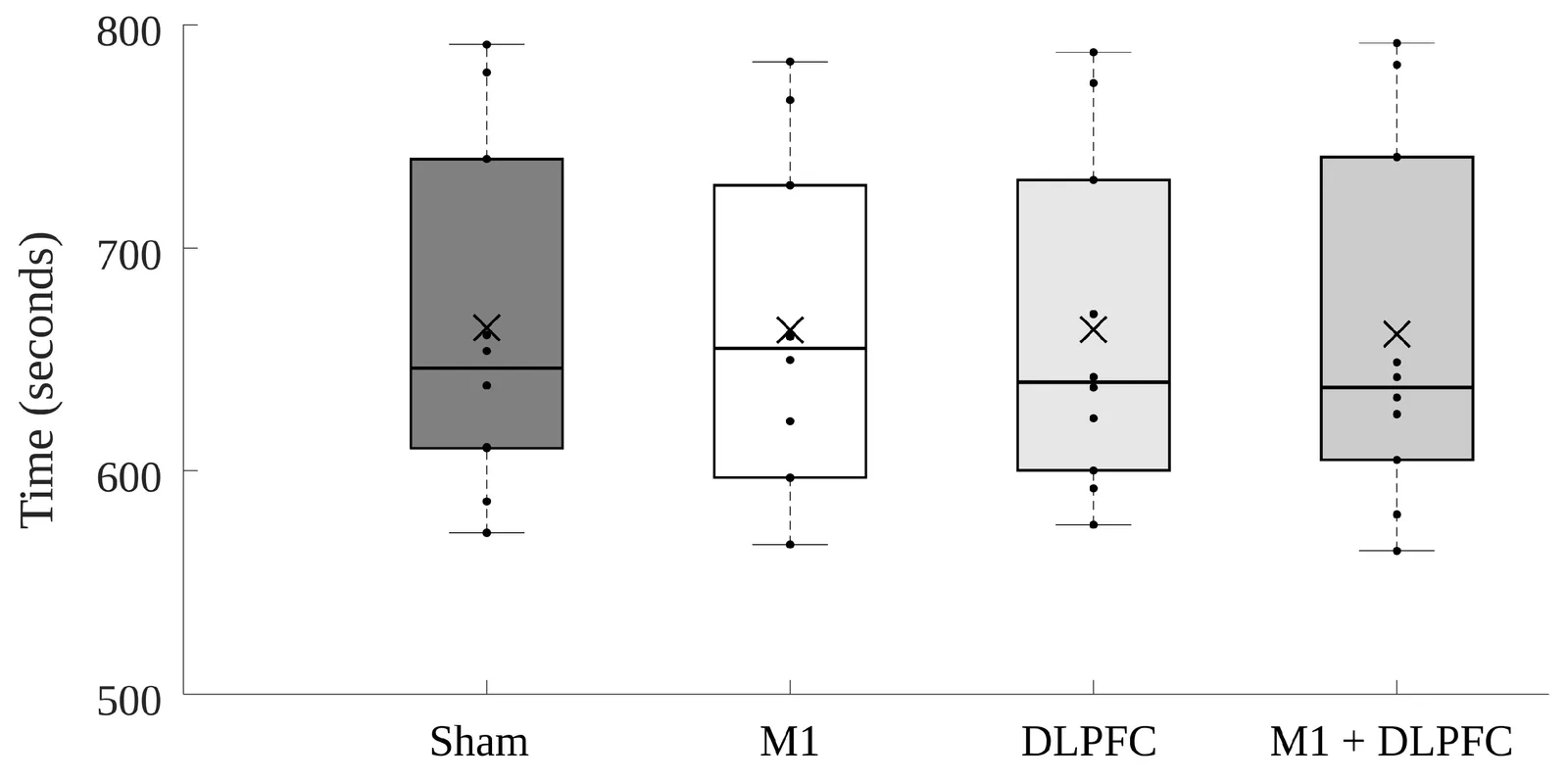
Total time (in seconds) to complete the 3000 m time-trial run across the four stimulation conditions: M1, DLPFC, Real, and Sham. Box plots show the group median as the horizontal line inside the box. Individual runner data are shown as black dots. The mean is represented by an X. No significant differences were observed between conditions.

The average time to complete the 3000 m time-trial run across all conditions was 10:19 (minutes: seconds; SD = 32 s) for males and 12:46 (SD = 25 s) for females. The average intersession variability was 7 s for males and 8 s for females. Total running time was similar across the four conditions (F = 0.21, *p* = 0.889, η^2^_p_ = 0.02), as seen in Figure 3. However, the sham condition produced the slowest average time of 11:04. The M1 only and DLPFC only conditions were faster than sham on average by 1 s (M1 only: d = 0.08; DLPFC only: d = 0.10) and the M1 + DLPFC condition was faster than sham on average by 3 s (d = 0.30). At the individual level, six of ten runners completed the M1 condition faster than their personal sham session, six of ten runners completed the DLPFC condition faster than sham and seven of ten runners completed the M1 + DLPFC condition faster than sham (Table 2), indicating that the group level trend of faster times with both M1 and DLPFC was shown in the majority of participants.

On average across all conditions, runners completed the time-trial 5.5% slower than the running time predicted by their $\overset{\cdot }{V}$O_2_max. No significant difference was found between conditions when comparing the predicted time to the runner’s actual time in each condition (F = 0.34, *p* = 0.798, η^2^_p_ = 0.04).

**Figure 4:**
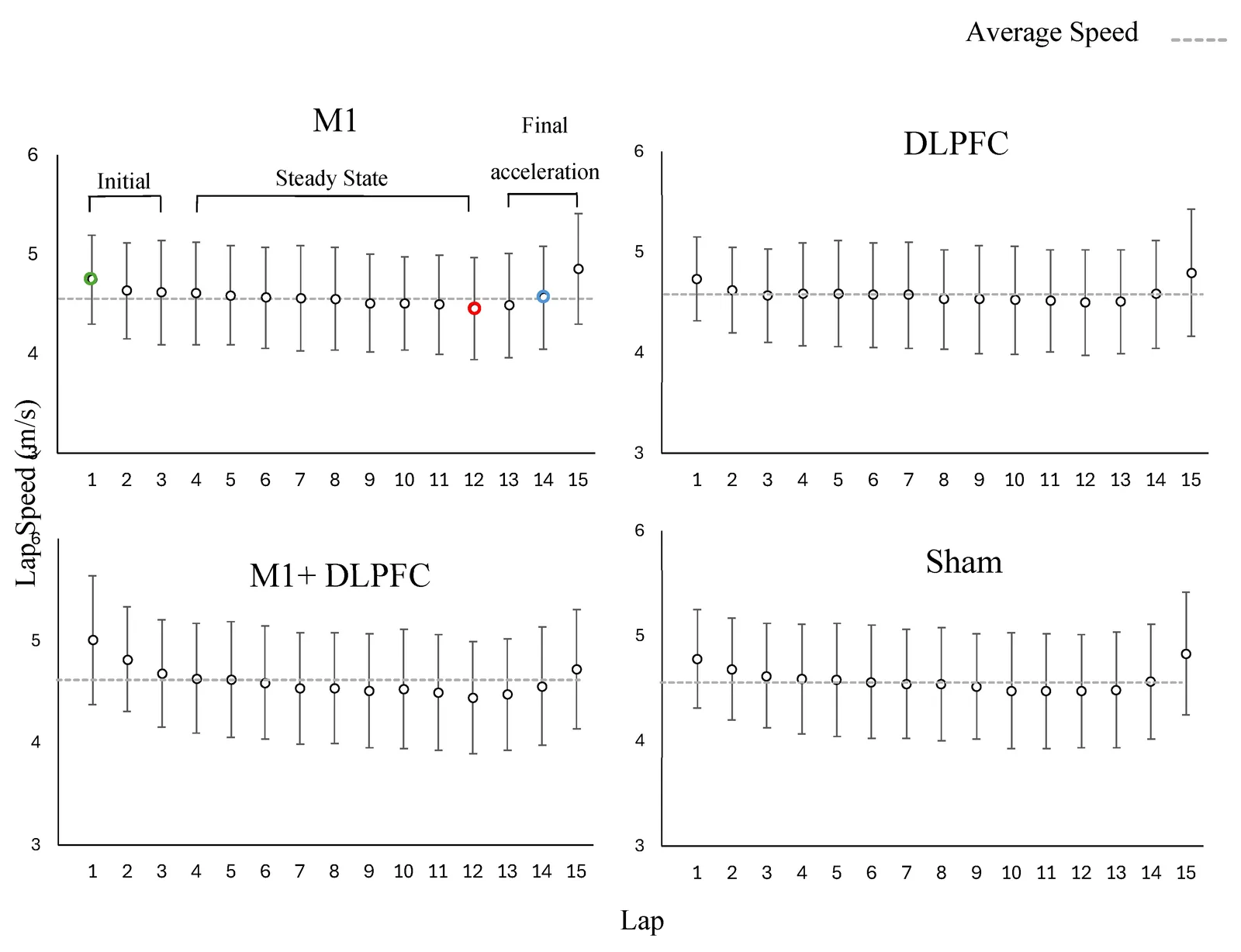
Lap speed (m/s) across 15 laps for each stimulation condition: M1 only, DLPFC only, M1 + DLPFC, and Sham. Data points represent the mean speed for all runners for each lap and error bars show standard deviation. The dotted line represents the average speed across all laps within a condition. The three phases of the run are shown on the M1 graph. The initial phase (laps 1–3) begins at the initial lap circled in green. The steady state lap goes from lap 4 until the slowest lap—lap 12, which is circled in red. The final acceleration phase is laps 13–15 with the final acceleration lap circled in blue.

As shown in Figure 4, runners followed typical pacing strategies observed in a 3000 m time-trial run [35] where they showed the fastest speed in the initial lap at an average speed of 4.81 m/s. They maintained a steady pace for most of the time-trial run with the slowest lap occurring at lap 12 at an average speed of 4.46 m/s (Lap 1 > Lap 12; Sham: t = 2.57, *p* = 0.015, d = 0.38; M1 only: t = 2.63, *p* = 0.014, d = 0.36; DLPFC only: t = 2.26, *p* = 0.025 m, d = 0.32; M1 + DLPFC: t = 2.61, *p* = 0.014, d = 0.68). Lap 12 was considered the end of the steady state phase. Then, runners increased to an average speed of 4.80 m/s for the final lap. There was a significant interaction between condition and lap for lap speed (F = 3.50, *p* = 0.047, η^2^_p_ = 0.28), showing that the M1 + DLPFC condition was significantly faster than the DLPFC only condition (+0.17 m/s, *p* = 0.043, d = 0.74) in the initial lap, and that the sham condition was significantly faster than the M1 + DLPFC condition in the final acceleration lap (+0.11 m/s, *p* = 0.031, d = 0.80).

Significant differences were found between the percent change across phases and conditions (χ^2^ = 48.99; *p* < 0.001); however, no significant differences were found when comparing conditions in each phase. Across all conditions, speed consistently decreased in the initial phase (Sham: −3.32%, M1 only: −2.87%, DLPFC only: −3.48%, M1 + DLPFC: −5.99%) and steady state phase (Sham:−2.59%, M1 only: −3.39%, DLPFC only: −1.80%, M1 + DLPFC: −3.99%), and increased in the final acceleration phase (Sham: +7.71%, M1 only: +8.36%, DLPFC only: +6.35%, M1 + DLPFC: +5.62%).

### 3.2. Perceived Exertion

The average RPE for lap 15 across all conditions was 19.5, indicating that runners finished the time-trial run near maximal exertion (Table 3). Average RPE significantly increased from lap 3 to 15 in all conditions (F = 122.98, *p* < 0.001, η^2^_p_ = 0.93). No significant differences were found by condition (F = 0.59, *p* = 0.628, η^2^_p_ = 0.06) or by the interaction of condition and lap (F = 0.75, *p* = 0.704, η^2^_p_ = 0.08).

### 3.3. Spatiotemporal Running Parameters

**Figure 5:**
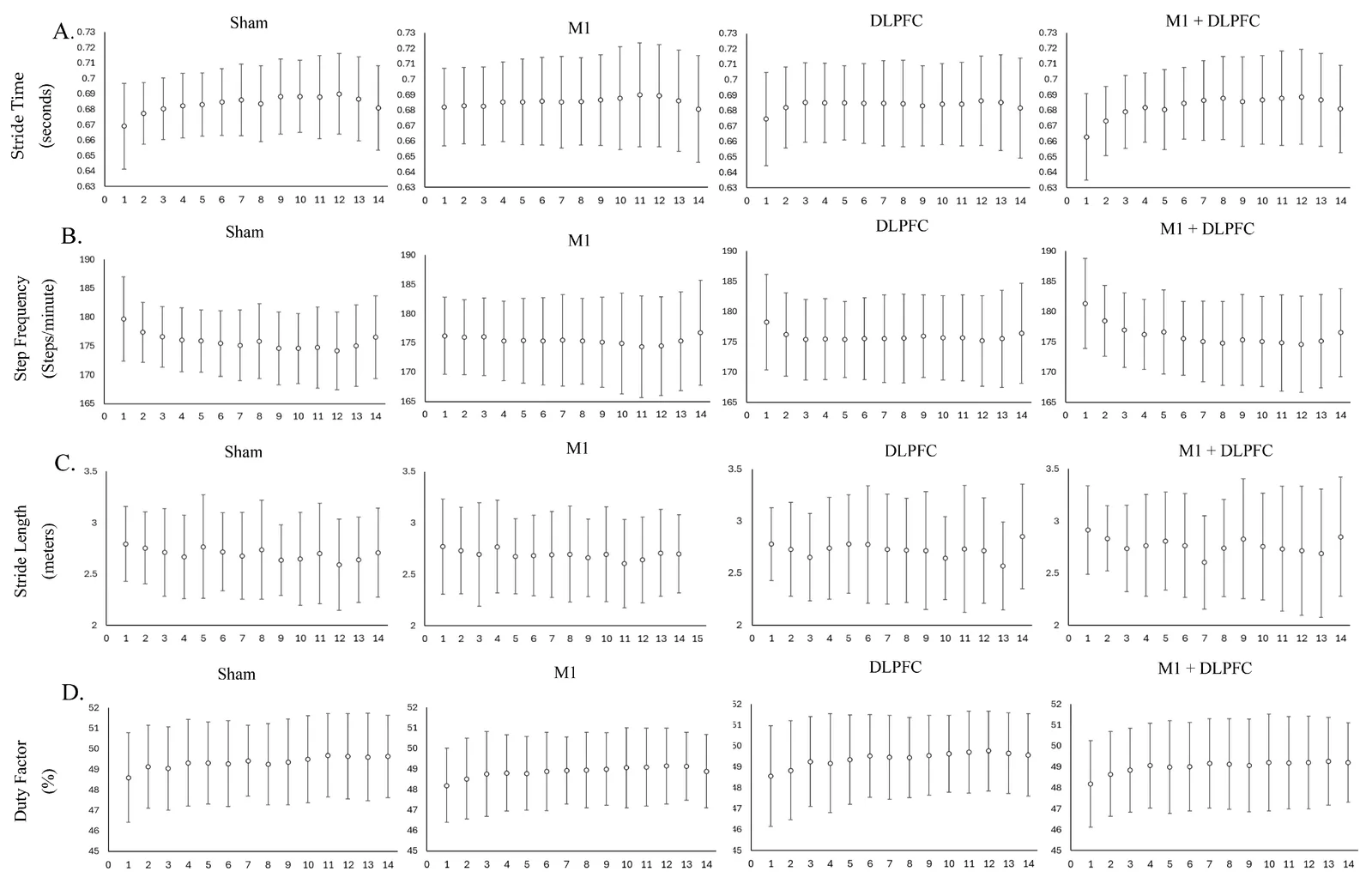
(**A**) Average ST across 14 laps for each stimulation condition: M1, DLPFC, M1 + DLPFC, and Sham, (**B**) Average SF across 14 laps for each stimulation condition: M1, DLPFC, M1 + DLPFC, and Sham, (**C**) Average SL across 14 laps for each stimulation condition: M1, DLPFC, M1 + DLPFC, and Sham, (**D**) Average DF across 14 laps for each stimulation condition: M1, DLPFC, M1 + DLPFC, and Sham.

There was a significant interaction between condition and lap for stride time (F = 3.58, *p* = 0.032, η^2^_p_ = 0.29), showing that the M1 + DLPFC condition had a shorter stride time compared to the M1 only condition in the initial lap (M1 + DLPFC: 0.66 s, M1 only: 0.68 s, *p* = 0.011, d = 1.00), as seen in Figure 5A. Significant differences were found between the percent change across phases and conditions (χ^2^: 34.89, *p* < 0.001), with the M1 only condition showing a significantly smaller percent increase in stride time in the initial phase compared to the DLPFC only (Z = 2.60, *p* = 0.009), M1 + DLPFC (Z = 2.60, *p* = 0.009) and sham (Z = 2.19, *p* = 0.028) conditions (Sham: +1.75%, M1 only: +0.11%, DLPFC only: +1.63%, M1 + DLPFC: +2.52%), and the DLPFC only condition showing a significantly smaller percent increase in the steady state compared to the sham condition (DLPFC only: +0.2%, sham: +1.1%, Z = 2.50, *p* = 0.013).

There was a significant interaction between condition and lap in SF (F = 3.61, *p* = 0.030, η^2^_p_ = 0.29), showing that the M1 + DLPFC condition had a higher SF compared to the M1 only condition in the initial lap (M1 + DLPFC: 182 steps/minute, M1 only: 176 steps/min, *p* = 0.011, d = 1.01), as seen in Figure 5B. Significant differences were found between the percent change across phases and conditions (χ^2^: 34.83; *p* < 0.01), showing a smaller percent decrease in the DLPFC only compared to the sham condition in the steady state phase (DLPFC only: −0.17%; sham: −1.07%; Z = 2.50, *p* = 0.013). At the individual level, seven out of ten runners showed both a shorter initial-lap stride time and a higher initial-lap step frequency in the M1 + DLPFC condition than in their own sham session, compared with five of ten in the DLPFC only condition and two of ten in the M1 only condition, indicating that an early response to combined stimulation was present in the majority of runners.

No significant interaction was found between condition and lap in SL (F = 0.59; *p* = 0.739, η^2^_p_ = 0.06), as seen in Figure 5C. Significant differences were found between the percent change across phases and conditions (χ^2^: 38.35, *p* < 0.001), showing a significantly smaller percent decrease in the DLPFC only condition compared to the M1 only condition in the steady state phase (M1 only: −4.35%, DLPFC only: −0.73%, Z = 2.09, *p* = 0.037), and a significantly larger percent increase in the DLPFC only condition compared to the M1 only (Z = 2.40, *p* = 0.017) and the sham condition (Z = 2.40, *p* = 0.017) in the final acceleration phase (Sham: +2.61%, M1 only: +0.09%, DLPFC only: +10.92%, M1 + DLPFC: +6.51%).

No significant interaction was found between condition and lap in DF (F = 0.45, *p* = 0.716, η^2^_p_ = 0.05), as seen in Figure 5D. No significant interactions were found between conditions and phase in DF (F = 0.93, *p* = 0.482, η^2^_p_ = 0.09). Across all conditions DF increased in the initial phase (Sham: +0.91%, M1 only: +1.13%, DLPFC only: +1.47%, M1 + DLPFC: +1.38%) and steady state phase (Sham: +0.65%, M1 only: +0.71%, DLPFC only: +1.25%, M1 + DLPFC: +0.28%). In the final acceleration phase M1 only, DLPFC only and M1 + DLPFC conditions showed minor decreases (M1 only: −0.47%, DLPFC only: −0.16%, M1 + DLPFC: −0.09%) and the sham condition showed a minor increase (Sham: +0.06%). Quantitative values of the parameters plotted in Figure 5 are reported in Table 4.

### 3.4. Variability of Spatiotemporal Parameters

Unlike speed, the variability of ST, SF, SL, and DF fluctuated around a stable mean, with small and irregular increases and decreases throughout the run. There was no significant interaction between conditions and lap for CV of any of the spatiotemporal parameters. (ST: F = 0.43, *p* = 0.854, η^2^_p_ = 0.05; SF: F = 0.44, *p* = 0.846, η^2^_p_ = 0.05; SL: F = 0.43, *p* = 0.854, η^2^_p_ = 0.05; DF: F = 1.18, *p* = 0.338, η^2^_p_ = 0.12). There was also no significant interaction between conditions and phase in the change in variability in any of the spatiotemporal parameters. (ST: χ^2^ = 7.25, *p* = 0.779; SF: χ^2^ = 7.57, *p* = 0.751; SL: χ^2^ = 7.25, *p* = 0.779; DF: F = 0.97, *p* = 0.452, η^2^_p_ = 0.09).

## 4. Discussion

This study examined the effects of non-invasive brain stimulation on running performance. It specifically measured the changes induced by increased excitability of the primary motor cortex and dorsolateral prefrontal cortex on running time, speed, perceived exertion, and spatiotemporal parameters and their variability during a 3000 m time-trial run. We hypothesized that the combined stimulation of the primary motor cortex and dorsolateral prefrontal cortex would enhance running performance by mitigating the decrease in neural drive due to exercise-induced fatigue and by alleviating pain perception which impacts motivation and cognitive control. These alterations would be seen in running time, speed, perceived exertion, and spatiotemporal variability. Stimulation of the M1 only was expected to show beneficial effects on running time and speed, and stimulation of the DLPFC only was expected to show beneficial effects on RPE and spatiotemporal variability. Our hypotheses were partially supported, with the M1 + DLPFC showing the shortest running time and the fastest speeds in the initial lap across all conditions. However, the M1 only showed no benefit in running time or speed compared to DLPFC only or sham condition. RPE and spatiotemporal variability were not affected by any type of brain stimulation with no differences between conditions as expected.

The M1 + DLPFC condition recorded the fastest running time overall, nearly three seconds faster than the sham condition, and about one second faster than stimulation of either area alone. In competitive running, even just three seconds could determine podium placement. However, three seconds is within the typical intersession variability for our sample, indicating that other external or internal factors, outside of brain stimulation, could have caused this difference. Therefore, the difference should be treated as a descriptive trend to be tested in a larger sample, rather than as evidence of a definitive performance benefit. It is important to note that higher-level runners are expected to show more minor improvements, with Barbosa et al. [36] reporting average improvements of about seven seconds for 3000 m world record holders from the season they set the record to the previous season. Comparatively, the one other study that assessed running performance on a track after increasing excitability found that the stimulation group was 69 s faster than the sham group in a 5000 m run [8]. However, this study used different runners in each group, further supporting the idea that there were pre-existing differences between groups. When investigating running performance, smaller effects in the same runner can often be more meaningful than larger effects between different runners.

The M1 + DLPFC condition generated the highest running speed during the first lap compared to the other conditions and was significantly faster compared to the DLPFC only condition, as expected. However, speed was not significantly increased in the M1 only condition, contradicting our hypothesis. Notably, RPE recorded after the initial phase, and throughout the run, was not significantly different between conditions, similar to previous running studies [8,9,10,11]. This may indicate that increased neural drive caused from increasing excitability in the M1 is less meaningful with no concomitant increasing excitability in the DLPFC, leading to enhanced task prioritization. However, it should be noted that task prioritization was not directly measured in this study and should therefore be considered with caution. Since the central nervous system continuously integrates multiple signals to determine exercise intensity [37], it is possible that enhancing both motor and cognitive control signals at the same time is necessary to translate into meaningful performance improvements. This demonstrates how multiple metrics of running performance recorded at different time points may reveal subtler differences than the running time alone. The stability of RPE when examined in conjunction with other metrics like lap speed revealed increased physical performance with no increase in perceived fatigue. The initial increased speed provided further insight on how total running time was affected. The higher running velocity recorded at the start of the time-trial was sufficient to produce the best running time trial following the M1 + DLPFC stimulation, even though the M1 + DLFPC condition showed the greatest decrease in speed in both successive phases (i.e., the steady state phase and the final acceleration phase). This pattern of a fast start then larger decline indicates there may be a pacing trade-off. Runners in the M1 + DLPFC condition may have capitalized on the early increase in excitability by running faster early, at the cost of a steeper decline later and a significantly slower final lap relative to sham. This would suggest that combined M1 + DLPFC stimulation altered the runners’ pacing behavior instead of physiological factors. We did not directly test for a trade-off and therefore, this remains speculative until testing with a larger sample size is performed. This increased running velocity following the M1 + DLPFC stimulation disappeared during the steady state phase of the time-trial with no significant differences in RPE or lap speed after the initial phase. This may suggest that the increase in cortical excitability of both M1 and DLPFC was not sufficient to last throughout the entirety of the time-trial run and overcome the effects of fatigue experienced during endurance exercise. Additionally, runners did not start the time-trial for at least 12 min with the time to arrive at the track and complete their warm-up. Although iTBS has been found to have lasting effects for 60 min, these effects have shown to peak at 15 min prior to beginning to decline back to baseline [38], possibly indicating that the runners experienced meaningful effects for the first three minutes of the run. This timing offers a possible explanation for why the effects of stimulation were detected in the initial lap only, as by the time runners got to the steady-state phase, the peak effects had likely already began to decline. Future studies may, therefore, consider performing a warm-up first and then begin the running time-trial immediately after the brain stimulation to test during the period of peak effectiveness, or explore ways to deliver TMS at the track to reduce time between stimulation and the onset of running.

Due to the lack of consensus in the literature regarding the magnitude of spatiotemporal parameters, no specific hypothesis was made for each parameter. In general, our results found increases in ST and DF and decreases in SF and SL between the first lap and the steady state lap, suggesting that in the earlier laps, the runner was not experiencing fatigue. Increasing excitability in both the M1 + DLPFC appeared to have an effect early in the run, with the lowest values for ST and DF and the highest values of SF and SL seen in the initial lap of the M1 + DLPFC condition. Like speed, ST, SF and SL then showed the greatest percent change in the M1 + DLPFC in the steady state phase, further supporting the idea that the effects of brain stimulation may only impact non-fatigued running or wear off soon after beginning the run. These changes in spatiotemporal parameters seemingly induced by increased excitability could be beneficial for some runners depending on their personal running style. Since parameters shifted in the same direction as in the increase in speed, this may reflect an early-phase change in gait pattern. However, even though the condition by lap interactions for ST and SF were significant in the initial lap, the absolute differences were small (0.02 s in ST and 6 steps/min in SF between M1 + DLPFC and M1 only conditions) and unlikely to be apparent to a runner or change their race strategy. Therefore, whether these results are competitively meaningful cannot be determined from this sample.

Contrary to our third hypothesis, none of the spatiotemporal parameters showed a significant change in variability across conditions or run phases, indicating that stimulation of the M1, DLPFC, or both did not influence gait consistency in this sample. The lack of effect that increasing excitability had on spatiotemporal parameter variability in our running study contrasts with findings in walking studies, where gait variability was reduced in healthy adults after stimulation of the DLPFC. This may be attributed to fundamental differences between walking and running mechanics. Running introduces a flight phase, which leads to increased variability, while walking includes a double support phase, which contributes to greater stability [39]. Increased excitability may enhance stability during walking’s double support phase, but its impact on the flight phase in running may be more limited. The other consideration that is important to note is that the reduced variability was seen during dual tasking [22]. Although there are cognitive processes involved in running related to optimal pacing strategies [40], runners were not performing a more demanding cognitive test such as a Stroop task. Therefore, the usefulness of increasing excitability in the DLPFC may lie in stabilizing walking patterns that are disrupted when the brain must simultaneously complete a straining cognitive task. In contrast, variability control, especially in our athlete participants, is sufficiently controlled without brain stimulation and does not leave a margin of improvement with stimulation.

Several limitations of this study should be noted. Due to challenges with consistency in health habits, recruitment and retention, the final sample size was small, consisting of ten runners. This sample included both males and females and all runners were analyzed together, even though there are known sex-differences in hormones, cortical excitability and response to iTBS [31]. As a result, some of the variability between conditions may be influenced by factors that vary by sex. While many studies include only male participants to minimize these differences, female runners were included so the findings could be generalized to a larger population of runners. However, this sample could not sufficiently address the current underrepresentation of female runners in the literature, as there were just three females in the sample, which did not allow for a meaningful analysis of sex-differences. Another consequence of the small, unevenly distributed sample is the comparatively large vulnerability to type 2 errors. A final limitation to note is that the analysis was performed solely on the straight running segment that was closest to the APDM receiver to ensure data consistency and quality. However, excluding the curved sections limited the running data and the gait cycles available to analyze for each lap, which could potentially have affected the results. Further differences could have been observed in the turning segments that were not accounted for in this analysis.

## 5. Conclusions

Our findings suggest that acute iTBS stimulation of both the M1 and DLPFC in endurance-trained runners shows potential for improving overall time and increasing speed, ST, and SF during the initial phase of a 3000 m time-trial. Since this study was exploratory in nature due to the limited sample size, these findings should be viewed as preliminary, rather than as definitive evidence of improvement in performance. The use of wearable IMUs enabled the continuous assessment of spatiotemporal parameters throughout the run, providing insight into adaptations that were not fully reflected by completion time alone. Faster lap speeds in the initial phase without an increase in RPE may suggest that stimulation of both the M1 and DLPFC has the ability to enhance motor output and efficiency, allowing for greater speed without a corresponding increase in perceived effort. However, because direct measures such as oxygen cost would be needed to confirm this possibility, this interpretation needs to be considered with caution. Future research should investigate testing within the first 15 min after stimulation to maximize its effects or explore stimulation of other brain regions, such as the cerebellum, that may influence variability, balance, and coordination. These findings highlight the potential of increasing excitability in both the M1 and DLPFC to enhance multiple aspects of running performance and demonstrate the value of sensor-based biomechanical assessment for evaluating responses to neuromodulatory interventions. However, further studies using similar protocols and larger samples are needed before definitive conclusions can be made regarding their efficacy.

## Figures and Tables

**Table 1:** Description of runner characteristics.

ID	Sex	Age	Height (cm)	Weight (kg)	Edinburgh Handedness Score	Waterloo Footedness Score	Specialization (Distance)	Years of Training	Training Volume per Week (km)	$\overset{\cdot }{V}$O_2_max (mL/kg/min)	Best 5 km (min)	Best 10 km (min)	Mercier Performance Index
RUN_01	M	26	183	72	90	17	Middle	12	50	56.5	N/A	33:25	574
RUN_11	M	21	183	65	100	15	Middle	7	60	61.6	15:30	32:45	610
RUN_03	M	22	191	64	100	1	Middle	9	65	75.4	14:45	N/A	703
RUN_06	M	37	172	75	80	18	Long	10	55	57.3	20:00	43:00	195
RUN_20	M	29	172	71	80	0	Long	10	100	64.1	N/A	34:00	554
RUN_05	M	22	182	70	100	4	Long	3.5	60	64.7	19:34	39:34	226
RUN_17	M	31	167	70	100	6	Long	3	50	73.4	17:00	35:00	447
RUN_25	F	18	172	70	79	8	Long	2	35	47.3	20:00	50:00	513
RUN_16	F	41	167	51	100	5	Long	20	50	52.2	20:00	43:00	513
RUN_15	F	20	162	46	100	15	Long	4	32	56.2	22:00	N/A	395
Average	N/A	27	175	65	93	9	N/A	8	56	60.9	18:36	39:37	473
SD	N/A	8	9	10	10	7	N/A	5	19	8.9	2:32	6:12	162

Abbreviation: N/A, not available.

**Table 2:** Mean (SD) completion time for the 3000 m time-trial by stimulation condition, and number of runners individually faster than their own sham time.

Condition	Mean Time (min:ss)	SD (s)	Faster than Sham (*n*/10)
M1	11:03	74.0	6
DLPFC	11:03	75.8	6
M1 + DLPFC	11:01	81.5	7
Sham	11:04	79.0	—

**Table 3:** Average (SD) rate of perceived exertion by lap and condition.

Condition	Lap				
	3	6	9	12	15
M1	11.4 (3.3)	14.2 (1.5)	16.2 (1.0)	17.6 (0.8)	19.4 (0.7)
DLPFC	12.3 (1.4)	14.4 (1.2)	16.2 (1.0)	18.1 (1.1)	19.5 (0.7)
M1 + DLPFC	12.1 (1.7)	14.8 (1.4)	16.5 (1.2)	17.9 (1.0)	19.7 (0.5)
Sham	12.4 (2.3)	14.6 (1.8)	15.9 (1.6)	17.5 (1.2)	19.3 (0.5)

**Table 4:** Mean (SD) spatiotemporal running parameters for the three comparison laps used to define the initial, steady-state, and final acceleration phases, by stimulation condition (*n* = 10).

Parameter	Lap	M1	DLPFC	M1 + DLPFC	Sham
Stride time (s)	1	0.68 (0.03)	0.67 (0.03)	0.66 (0.03)	0.67 (0.03)
	12	0.69 (0.03)	0.69 (0.03)	0.69 (0.03)	0.69 (0.03)
	14	0.68 (0.03)	0.68 (0.03)	0.68 (0.03)	0.68 (0.03)
Step frequency (steps/min)	1	176.21 (6.57)	178.26 (7.91)	181.36 (7.43)	179.68 (7.32)
	12	174.46 (8.45)	175.17 (7.46)	174.61 (7.95)	174.16 (6.74)
	14	176.73 (8.97)	176.43 (8.27)	176.54 (7.29)	176.53 (7.19)
Stride length (normalized)	1	2.77 (0.46)	2.78 (0.35)	2.91 (0.42)	2.79 (0.36)
	12	2.64 (0.42)	2.72 (0.51)	2.72 (0.62)	2.59 (0.44)
	14	2.70 (0.38)	2.86 (0.50)	2.85 (0.57)	2.71 (0.43)
Duty factor (%)	1	48.20 (1.81)	48.56 (2.41)	48.19 (2.06)	48.61 (2.18)
	12	49.14 (1.85)	49.76 (1.90)	49.21 (2.21)	49.64 (2.08)
	14	48.90 (1.78)	49.57 (1.96)	49.21 (1.89)	49.64 (2.01)

## Data Availability

Dataset available on request due to restrictions.

## Abbreviations

The following abbreviations are used in this manuscript:

AMT	Active motor threshold
CNS	Central nervous system
CT	Contact time
CV	Coefficient of variation
DF	Duty factor
DLPFC	Dorsolateral prefrontal cortex
EMG	Electromyography
FDI	First dorsal interosseous
FT	Flight time
IMU	Inertial measurement unit
iTBS	intermittent theta burst stimulation
M1	Primary motor cortex
MEP	Motor evoked potential
MRI	Magnetic resonance imaging
MVC	Maximum voluntary contract
NIBS	Non-invasive brain stimulation
RM-ANOVA	Repeated measures analysis of variance
RPE	Rating of perceived exertion
rTMS	Repetitive transcranial magnetic stimulation
SF	Step frequency
SL	Stride length
ST	Stride Time
TA	Tibialis Anterior
TBS	Theta burst stimulation
tDCS	Transcranial direct current stimulation
TMS	Transcranial magnetic stimulation
$\overset{\cdot }{V}$O_2_max	Maximal oxygen uptake
